# Drone-Based Water Level Detection in Flood Disasters

**DOI:** 10.3390/ijerph19010237

**Published:** 2021-12-26

**Authors:** Hamada Rizk, Yukako Nishimur, Hirozumi Yamaguchi, Teruo Higashino

**Affiliations:** 1 Graduate School of Information Science and Technology, Osaka University, Osaka 565-0871, Japan; nishimura@mc.net.ist.osaka-u.ac.jp (Y.N.); h-yamagu@ist.osaka-u.ac.jp (H.Y.); higashino@ist.osaka-u.ac.jp (T.H.); 2 Computer and Automatic Control Department, Faculty of Engineering, Tanta University, Tanta 31733, Egypt

**Keywords:** drone-based vision, emergency recovery, flood disaster assessment, water level detection

## Abstract

Japan was hit by typhoon Hagibis, which came with torrential rains submerging almost eight-thousand buildings. For fast alleviation of and recovery from flood damage, a quick, broad, and accurate assessment of the damage situation is required. Image analysis provides a much more feasible alternative than on-site sensors due to their installation and maintenance costs. Nevertheless, most state-of-art research relies on only ground-level images that are inevitably limited in their field of vision. This paper presents a water level detection system based on aerial drone-based image recognition. The system applies the R-CNN learning model together with a novel labeling method on the reference objects, including houses and cars. The proposed system tackles the challenges of the limited and wild data set of flood images from the top view with data augmentation and transfer-learning overlaying Mask R-CNN for the object recognition model. Additionally, the VGG16 network is employed for water level detection purposes. We evaluated the proposed system on realistic images captured at disaster time. Preliminary results show that the system can achieve a detection accuracy of submerged objects of 73.42% with as low as only 21.43 cm error in estimating the water level.

## 1. Introduction

Every year, floods are caused by typhoons and torrential rains worldwide, causing a lot of damage. For instance, after heavy rains hit southeastern Brazil in January 2020, 44 people died, and 13,000 people were affected by the floods [1]. Hurricane Harvey hit southeast Texas and southwest Louisiana in 2017. As a result, the loss of houses due to water damage from the flood has risen to over USD 25 billion [2]. Flood events are expected to become more frequent and damaging due to global climate change [3,4]. Thus, an automated system to detect and analyze the damage arising from such disasters is vital to relieve such a dramatic loss and damage. This enables a right-on-time response and efficient distribution and management of limited resources and rescue teams and disaster supply kits, reducing the risk of human fatalities.

To quantitatively gauge the water level, some dedicated sensors can be installed in areas susceptible to flooding, e.g., riverine areas. Several sensor-based systems, e.g., [5,6] have been proposed for measuring pressure, bubble, floating, etc. However, these systems suffer from several challenges that hinder their real-world adoption due to the limited area covered by fixed sensors and the high cost of installing and maintaining a large number of distributed sensors.

On the other hand, several computer vision approaches have been proposed for understanding and estimating damages in flood situations. Witherow et al. [7] propose an image processing pipeline for detecting water-level extent on inundated roadways from image data captured and generated by mobile consumer devices (e.g., smartphones). However, their approach requires images before the flood damages for identifying flooded areas using location-matched dry/flooded condition image pairs. Chaudhary et al. [8] proposed a system to predict the water level from social media pictures, which cannot provide a real-time assessment of the situation due to the late availability of images/information on social media. Vitry et al. [9] propose an approach that provides qualitative flood level trend information at scale from fixed surveillance camera systems. However, this approach is limited to specific locations where cameras are fixed. Although some studies analyze the flood situation on aerial images taken from remote satellites [10,11,12], the information extracted is on a macro-scale and hardly in real-time.

The aerial technology of drones (micro-scale) can provide information-rich visual data from the top view with wide coverage. In addition, the recent advancement of deep learning approaches provides better learning ability compared to traditional learning/vision techniques. Motivated by these advantages, we attempt to answer the following question: is it possible to estimate the water level of a flood incident based on aerial images?

In this paper, we present the design of a system that estimates water levels of submerged houses and cars from drone-based aerial information (top-view images). This is done by enabling the system to learn the inverse relationship between the water level and the visible parts of the submerged houses and cars. In particular, this is achieved in two stages. Initially, the proposed system employs a Mask Region-based Convolutional Network (Mask R-CNN) [13] to segment and extract objects (i.e., houses and cars) from the images used. Next, a VGG-16 network is trained to quantify the water level in the objects detected from the first stage.

To achieve highly accurate and robust detection, the proposed system needs to establish solutions for a number of challenges, including handling (1) blurring in the captured images, (2) data from multiple sources (e.g., drones or helicopters) captured at different altitudes, (3) the scarcity of labeled flood images (i.e., since disasters are uncommon), (4) enabling the considered networks to work well on top-view images on which they are not originally trained as well as avoiding over-fitting to training data. Therefore, we leverage different data augmentation methods to automatically increase the size of the training data and boost the model robustness to avoid over-fitting, automatic detection, and removal of blurred and misleading objects/images, as well as introduce an annotation strategy to identify the water-level based on the standard specifications of submerged objects. Thereafter, a transfer learning approach is applied to the pre-trained models using the small dataset, enabling these models to recognize objects and identify water-level from the top-view.

We implemented the proposed system leveraging realistic aerial images at flood disasters. Our results show that the system can achieve a detection accuracy of submerged objects of 73.42% with as low as only 21.43 cm error in estimating the water level. This highlights the promise of the proposed system as a robust and accurate water-level estimation solution for flood disasters.

Contributions: Our contributions are fivefold: (1) We propose a novel aerial images-based water level estimation system in flood disasters using the powerful learning ability of deep neural networks. (2) We extend the mask R-CNN model to detect houses as one of its target classes and boost car detection from top-view images captured by drones which were not considered in training the original version. (3) We reuse the structure of the VGG-16 neural network for water level estimation purposes. (4) We ensure the efficient adoption of deep learning models and their generalization through using data augmentation techniques and the fusion of images collected from different sources. (5) We experimentally evaluate the performance of the proposed approach demonstrating its capability to accurately detect the water level while covering a wider area using drones.

The rest of this paper is structured as follows. In Section 2, we discuss the recent state-of-the-art techniques relevant to the work carried out in this paper. Section 3 presents in detail the methodology of the proposed system. In Section 4, we evaluate the system performance. Finally, we conclude the paper in Section 5.

## 2. Related Work

For detecting and assessing flood damage situations, many processing techniques are applied to various kinds of data, for instance, sensor modules [5,6], surveillance cameras [9,14], and crowdsourcing images on social networks [7,8]. This section introduces the general ideas of these state-of-the-art approaches.

### Flood Damage Detection and Assessment

Installed dedicated sensors can directly measure the water level where pressure, bubble, floating, and non-contact radar ray are often used [5,6,15,16,17,18]. The self-calibrated water-level gauge, proposed in [5], can achieve a wide-range measurement, up to 150 m, with only a millimeter-scale error. The sensing devices are expected to be placed in high-risk locations of flood disasters, usually referring to rivers and their environs. Marin-Perez et al. [6] have specially designed and developed a long-life device for measuring and forwarding the water level information attached with the localization data to the base station via wireless communication (i.e., Bluetooth). The devices come with low power consumption, low manufacturing cost, and only a minor impact on the natural environment. The approach proposed in [15] leverages wireless sensor networks for flood monitoring. That approach captures water level data using a random block-based sampler and a gradient-based compressive sensing. Flood level estimation based on a water level sensor and a pressure gauge is proposed in [16]. This was done leveraging a 2-class neural network to predict flood status according to pre-defined rules.

Meanwhile, some exploit the existing infrastructure, such as surveillance systems, to identify the flood situation. Shi-Wei et al. [14] make use of surveillance camera systems in targeting areas (i.e., riverside) and leverage a segmentation technique in computer vision for detecting flood disasters. Segmentation is a technique used to remove the surrounding objects from the geographical background and separate intrusive objects in the frame. They adopt a region-based segmentation approach for automatically estimating the flood risk of the observed area captured by the surveillance cameras. Similarly, Vitry et al. [9] propose a method to detect a change (trend) of water level from video streams acquired by generic surveillance cameras. The method computes the water level change from the difference of flood-covering pixel ratio between the connecting frames in the video. They apply *U-net* [19], a deep convolutional neural network (DCNN) for image segmentation for recognizing flood-covering pixels on each frame. Although the ratio of flooded pixels from DCNN correlates to the water level up to 75% on average using Spearman’s rank correlation coefficient measurement [20], the method cannot provide an absolute water level value. Convolutional neural networks are trained to detect the water level using a synthetic ground-level image dataset in [21]. FloodGAN is a deep convolutional generative adversarial network proposed in [22]. The network learns to generate 2D inundation predictions of pluvial flooding caused by nonlinear spatial heterogenic rainfall events. A transfer learning technique is investigated in [23] to perform water semantic segmentation and water level prediction using river camera-captured images.

On the contrary, relying on the location flexibility of mobile device users, a crowdsourcing-based flood damage assessment has been investigated so far. Witherow et al. [7] propose a method to estimate the water-covering area during floods using roadway images captured by smartphones and driver recorders. The benefit of this system is that the covered area can be large as more users participate. Still this system requires images in both dry (i.e., as reference) and flooded conditions at the same location for extracting the difference between them with a given threshold value. The efficacy of Camera Sensor (CS) for water level monitoring is investigated in [24] at varying operating and image capturing conditions e.g., varying tilting angles and distances.

Social media images posted by crowds have recently been used for flood level detection. Chaudhary et al. [8] adopt Mask R-CNN [13] for estimating the number and water level of submerged objects like persons, cars, buses, and bicycles in the ground-level images gathered from the social media. MS COCO dataset [25] with manually annotated water level is used to train the model to identify the water level in eleven discrete levels.

In [26], an approach is proposed for estimating the water level from social media images using multi-task learning. The flood estimation is defined as a per-image regression problem and combines it with a relative ranking loss of multiple images to facilitate the labeling process. Although this approach reduces the annotation overhead, it provides a coarse-grained estimation accuracy of the water level. Additionally, this approach can work only in the region where images are captured limiting its universal adoption. Image classification of flood-related images collected from social media platforms is adopted in [27] to discriminate between three general flood severity classes (i.e., not flooded, water level below 1 meter, and water level above 1 meter). Two convolutional neural network architectures were adopted for this DenseNet [28,29], EfficientNet [30] and attention-guided convolutional model [31]. However, this approach provides a rough estimate of the water level. On the other hand, the technique in [32] leverages both text and images posted on social media for disaster damage assessment. These social media images (crowdsourcing-based images) are usually taken from the ground, which are available in adequate numbers and are visually clear with high resolution. Meanwhile, aerial images have a lot of limitations in both the number and quality. In addition, a public dataset of objects (houses, cars, etc.) cannot directly be used for model training as the shooting angles are very different.

## 3. Drone-Based Flood Damage Assessment

### 3.1. System Overview

**Figure 1:**
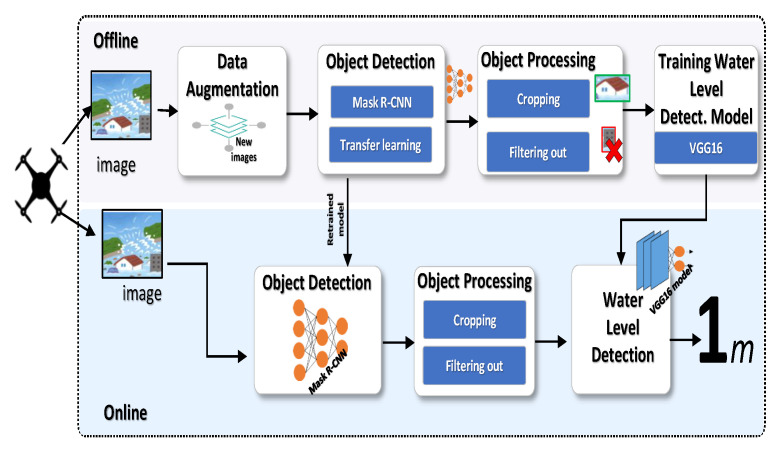
System architecture.

Figure 1 shows the system architecture. The proposed system has two stages: an offline stage and an online stage. The system is initialized in the offline stage by collecting the required drone-captured images. These images represent different flood levels as well as images without any flood in the areas of interest. Due to the scarcity of images in unusual situations like flood disasters, the collected images are further processed by the Data Augmentation module to synthetically generate new images enabling the efficient application of deep learning techniques. Then the system builds an Object Detection model using R-CNN by re-training the network on the available images. This model is responsible for classifying and segmenting objects in the drone-captured images, including houses and cars. Based on the information obtained from each image, the system uses an Object Processing module to decide whether to consider or filter out the objects within the image. After that, the system builds and trains a Water Level Estimation model for identifying the water level from the objects detected in each image.

During the online phase, the images captured by a drone will be forwarded to the Object Detection model, which is trained in the offline stage, for identifying and locating objects in the scene. These objects are fed to the Water Level Estimation to estimate the water level in the scene.

### 3.2. Dataset Preparation

The goal of this module is to prepare a dataset for training and evaluating the system’s deep models. The dataset consists of top-view images of the area of interest taken during a flood. Each image includes submerged objects (i.e., houses and cars) along with water-level annotation and has been prepared by the following steps: (1) image collection, (2) image selection, and (3) annotation.

#### 3.2.1. Image Collection

The top-view images are captured by drones during the disaster. Due to the rare occurrence of floods, we also gathered some aerial images from the Internet taken during the previous disasters by helicopters or from the roofs of tall buildings. The latter images are only considered in the offline phase, to compensate for the scarcity of images available for training the system’s models. Additionally, considering images from different sources with different factors (e.g., different photographed altitudes, image sizes, and resolutions) is believed to boost the generalization of the models when used in the online phase [33]. The dataset also includes aerial images in a normal situation (i.e., the water level is 0).

#### 3.2.2. Image Selection

This step is to filter out low-resolution or blurry images to avoid confusing the model. Specifically, one challenge in processing aerial drone imagery is the blur effect caused by camera movement during image acquisition. This can be caused by the abnormal flight movement of the drone (due to the wind) leading to misinterpretation of the data and degradation in the system accuracy. To combat this problem, the proposed system adopts an automatic method for blur detection [34] by obtaining a value representing how much an image is blurred. This is done by calculating the standard deviation of the image represents the difference between high pass filtered versions of both the original image and its low pass filtered one [34]. Images with high blurring factors or with low resolution are excluded as they may deceive the annotation process and thus the trained model [35,36].

#### 3.2.3. Annotation

**Figure 2:**
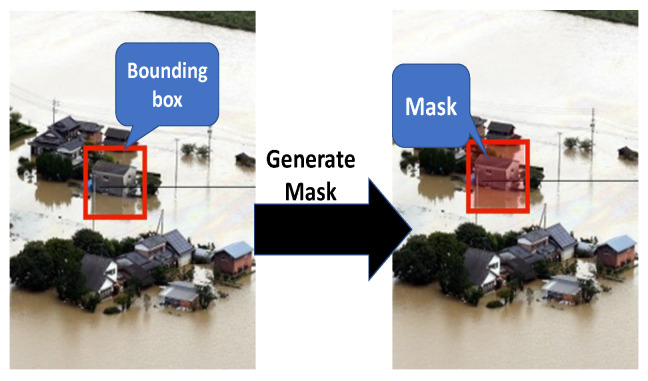
Mask generation.

Image annotation was carried out on *Supervisely*, image annotation, and data management platform. A label is given to each object of interest, which consists of a bounding box (a bounding box is an imaginary box that contains the target object as shown in Figure 2), a class (i.e., house and car), and a segmentation mask that covers only the pixels that belong to the object in the bounding box.

The annotation process is performed in three steps as described below. Firstly, the bounding boxes are attached to individual objects for each image together with their class annotations (i.e., house or car). Even though the mask of objects is required in order to train the Mask R-CNN model, it is not important for the proposed method. Thus, we assume that all pixels in the bounding box are the mask of the object covered by the box, as shown in Figure 2.

**Figure 3:**
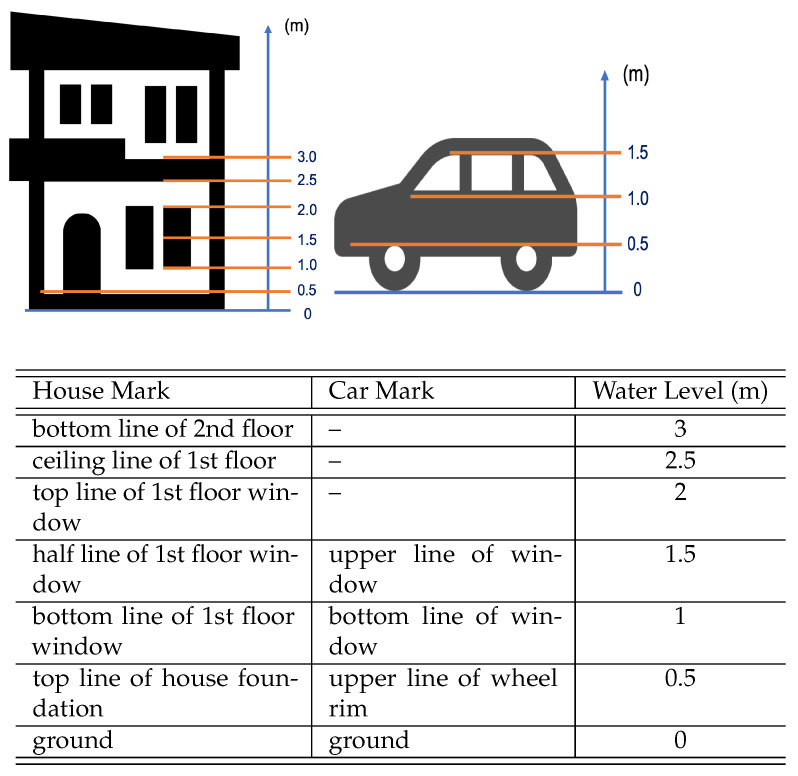
Standard dimensions of objects of interest.

Secondly, we annotate the water level for each object of interest (house or car) from 0 m to 3 m. Due to the challenge of obtaining an accurate ground truth of the water level, we captured training data from different sources including public news websites that contain both the flood information (i.e., the water level in the region) and flood images. Based on this information, we define specific discretized values for water-level as labels based on the standard dimensions of houses and cars shown in Figure 3. Specifically, the water level is up to 3 m for each house and up to 1.5 m for each car with a step of 0.5 m. For instance, the height of the bottom side of the window in a standard house is 1 m. Therefore, a house with water at this level is assigned the label “1 m”. It worth noting that water-level estimation is not an easy task due to occlusion by other houses/buildings as well as the camera angles from the sky. Therefore, water level values are assigned to the water levels of occluded objects based on the following rules:

(1) Houses and cars with the same level of roofs have the same elevation.
(2) Houses and cars with the same elevation are submerged to the same water level.
(3) In addition, buildings different from houses such as schools and hospitals as well as those which appear too small in the images are excluded as these images may deceive the detection model.

**Figure 4:**
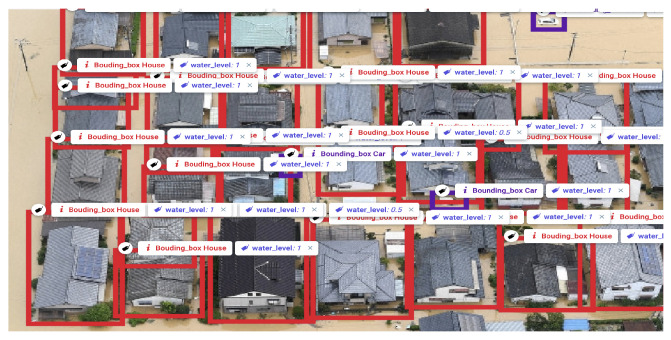
Labeling of houses and cars using Supervisely. The bounding boxes surrounding houses and cars are of the colors red and purple, respectively.

The output of the annotation process is the object surrounded by bounding boxes and labeled by their class and their corresponding water level, as shown in Figure 4. This data can be then stored in the form of a JSON file for further processing.

### 3.3. Data Augmentation

Image data augmentation is a set of techniques employed to synthetically generate artificial images given a small set of flood disaster images. Thus, data augmentation increases the training set volume enabling the effective utilization of deep learning and combatting the class imbalance problem. Moreover, data augmentation is vital in improving the model generalization ability by introducing new images covering different operational scenarios [33]. The proposed system adopts different image augmentation techniques, including flipping, random-rotating, noise-adding, brightness-adjusting, and zooming. Specifically, we employ horizontal and vertical flip augmentation where image columns and rows of pixels are reversed, respectively. This boosts the learning ability of the models to detect houses or cars from aerial photographs in any direction. On the other hand, rotation augmentation randomly rotates the image clockwise by a given number of degrees, which emulates different view angles of the drones, while noise-adding introduces random noise to the original images to generate a synthetic low-quality version of the image. This enables the model to work even with low-quality images, e.g., in rainy situations. The brightness of the images is also augmented by randomly darkening or brightening the available images. This generates new images, allowing the system to perform consistently in different lighting conditions, e.g., day-light or moon-light. Random zoom augmentation is adopted to emulate different flying altitudes and thus boosts the model’s generalization ability. This is done by randomly zooming the image (and therefore the objects) in or out by either adding new pixel values around the image or interpolating pixel values, respectively.

**Figure 5:**
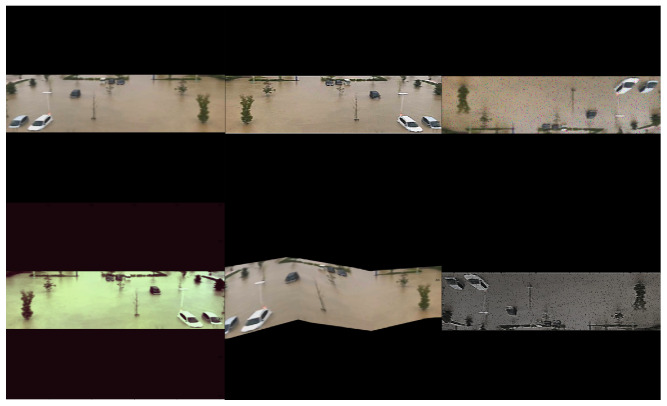
Examples of data augmentation results. We have applied flipping, adding the noise, and adjusting the brightness and color.

These techniques boost the learning ability of the deep models to cope with difficult situations that might occur in real-time. In this work, the data is augmented by *imgaug* [37], a python library for machine learning experiments. Figure 5 shows some examples of the artificial images as generated by data augmentation techniques.

Discussion: Excluding blurred images by the image selection module is only necessary at the offline pre-processing stage. This can be justified by noting that these images may lead to the incorrect annotation of the water level, which may negatively affect the trained model. On the other hand, we apply the noise-adding data augmentation technique to the selected and annotated images by introducing random noise to the original images. This is done to generate synthetic low-quality versions of the original image that contribute during training to improve the skills of the deep models to cope with blurred images that might be captured at the online stage (as proved in [33,38,39,40]).

### 3.4. Object Detection

**Figure 6:**
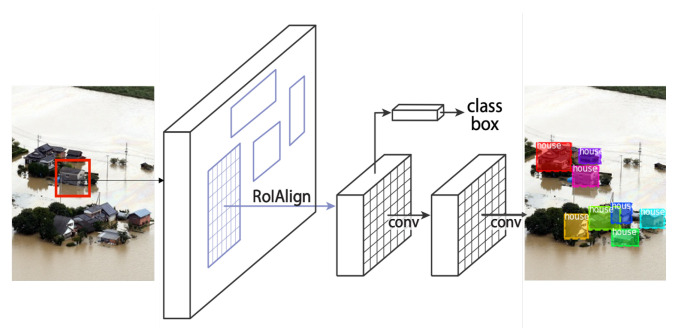
Model architecture of Mask R-CNN.

The object detection module is responsible for detecting and recognizing houses and cars given the top-view images captured by a drone. Without loss of generality, we adopt the Mask Region-Based Convolutional Neural Network (Mask R-CNN) [13], which is state-of-the-art architecture for object detection and segmentation. The network is built on Feature Pyramid Network (FPN) [41] and a ResNet [42]. ResNet is composed of some Backbone convolutional layers that extract simple features like lines and corners from the input image. After that, it runs through the Feature Pyramid Network (FPN) for extracting more concrete and complex features called feature maps. Then, Region Proposal Network (RPN) [43] is applied for marking a region of interest (RoI) that indicates the area that is likely to contain an object. Only the feature map on the highlighted RoI is considered at the output layers for locating and recognizing (classifying) objects (see Figure 6). The outputs from this network are the detected-and-classified objects (i.e., houses and cars) along with their bounding boxes.

**Figure 7:**
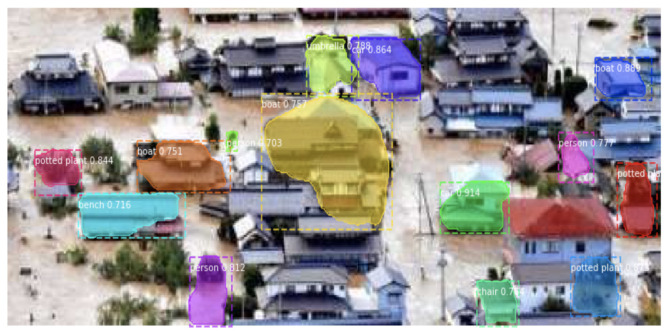
Recognition result of houses using pre-trained Mask R-CNN model. Houses are mislabeled.

The Mask R-CNN model is pre-trained using the CoCo dataset [25], and the model is provided in [44]. We have investigated how the pre-trained model performs when tested with top-view images that contain submerged houses and cars. Since the images contained in the CoCo dataset are taken from the ground level and do not include a class for houses, the detection accuracy severely drops. For instance, the majority of the houses are misclassified (e.g., classified as umbrellas and boats), as shown in Figure 7. To cope with this issue, we employ transfer learning using our top-view images. Since our dataset is small, retraining the entire model is not feasible. Additionally, the base convolutional part already contains features that are generically useful for classifying pictures. At the same time, the last layers are specific to ground-view image recognition and the objects on which the model was trained. Therefore, we retrain only the last layers of the pre-trained model to repurpose the feature maps learned previously. Our top-view images are pre-processed and augmented, as described in Section 3.2, before launching the training process.

In the model training process, there are five hyperparameters to be defined, including input image size, the number of RoI, training layer, learning rate, and epochs. Table 1 shows the selected values of these hyperparameters that maximize the performance of the proposed system. As images are collected from different sources, their sizes are unified before feeding them to the network. The large size of input images incurs a high computational time, whereas small size causes a loss of meaningful information in the original high-resolution images. Therefore, the size of the input layer has been selected carefully to avoid incurring high computational costs or information loss. This can be achieved by setting the input image size to 512×512 pixels (half of the images’ highest resolution in the dataset). In the aerial image, the expected minimum bounding box size of one object is about 4 × 4 pixels. Correspondingly, we set the number of RoIs to 128, which is the maximum number of objects that could appear in one image. The learning rate and the number of epochs are selected to ensure the best accuracy while avoiding overfitting. This can be obtained empirically at the learning rate and a number of epochs of 0.001 and 100, respectively.

### 3.5. Water Level Estimation

Estimating the water level from sky images of submerged surrounding objects is challenging. Given the unlimited number of cars and houses of variable shapes and heights, matching solutions are not possible. However, the annotation task leverages the information provided by our government about the standards of house elevation and structure as well as car description. From this information, the water level can be inferred from the submerged parts of the reference objects. Additionally, the proposed system leverages a learning-based solution to train a model for estimating the water level and ensuring its generalization ability. Deep Learning is currently considered the mainstream approach to Machine Learning in use today. Based on the Universal Approximation Theorem [45], Neural Networks could be considered as being capable of approximating any arbitrary function, provided they were suitably complex. Due to performance benefits obtainable from its use, deep networks have been shown to define the state-of-the-art performance in many problem domains (e.g., [46,47,48,49,50,51,52,53,54,55,56]), in some cases even outperforming humans on the same task.

**Figure 8:**
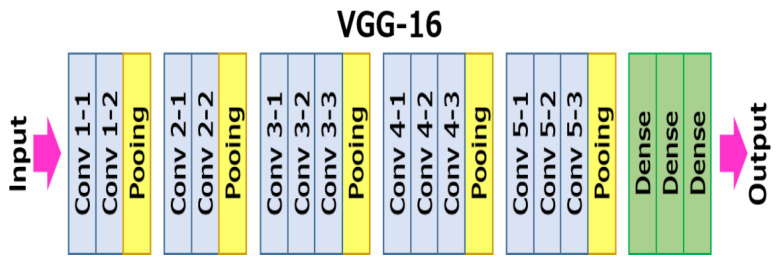
VGG16 model architecture [57].

The proposed system adopts the VGG16 network [57] for estimating the water level of each object. VGG-16 is a 16-layer convolutional neural network having an architecture shown in Figure 8. The whole network is composed of 13 convolutional layers, 5 max-pooling layers, and 3 fully connected layers. Only the 16 layers contain weights.

For training and validation purposes, in the offline phase, the car and house objects are cropped from the pre-processed images. These objects are cropped such that their areas should be slightly larger than their corresponding bounding boxes to cover object surroundings. This is done to facilitate the estimation of the water level around the cropped object. The cropping process is done automatically in the online phase from detected objects and their bounding boxes. We note that those images with a lot of “too small” objects, which are taken from too high altitude, are automatically filtered out. In particular, images whose average size of bounding boxes is smaller than the size of the image weighted by a threshold ratio α (0.005 in our system) are excluded. This is to ensure the consistency between the different images (in altitudes) and the information-richness of the detected objects.

We set 128×128 pixels for the input image size, which is the expected largest dimension of the cropped object image. Similar to the object detection module, all cropped object images are resized to the same dimension with the zero-padding technique. For the learning parameters, we use categorical cross-entropy as a loss function and Stochastic Gradient Descent (SGD) optimizer which outperforms other optimizers such as the Adam. The remaining parameters are decided empirically, aiming at both high validation accuracy and avoidance of over-fitting.

## 4. Evaluation

We evaluated the two primary functions of our system (i.e., object detection and water-level estimation) as follows. Forty-seven images of flood situations and five images of normal situations were used as a dataset.

For training the model, we used a machine with an Intel Xeon 2.20 GHz CPU and a Tesla P100 GPU with 16GB of RAM hosted on the Google Colaboratory platform.

### 4.1. Object Detection

The collected images contain 420 houses and 313 cars. 80% of these images are randomly selected for training and the remaining 20% are used for testing the object detection model (the Mask R-CNN). We have considered the mean Average Precision (mAP), precision, and recall as our target metrics. Average precision (AP) is a popular metric in measuring the accuracy of object detection. It represents the area under the precision-recall curve, which is a plot of the precision *p* (*y*-axis) and the recall *r* (*x*-axis) for different predicted confidence levels, that is, $AP=\int_{0}^{1}p\left(r\right)dr$. AP is 1.0 if the model achieves a perfect prediction. Accordingly, mAP is a mean of AP from all images, that is, $mAP=\sum_{i=1}^{N}AP_{i}$ where *N* is the number of images.

**Figure 9:**
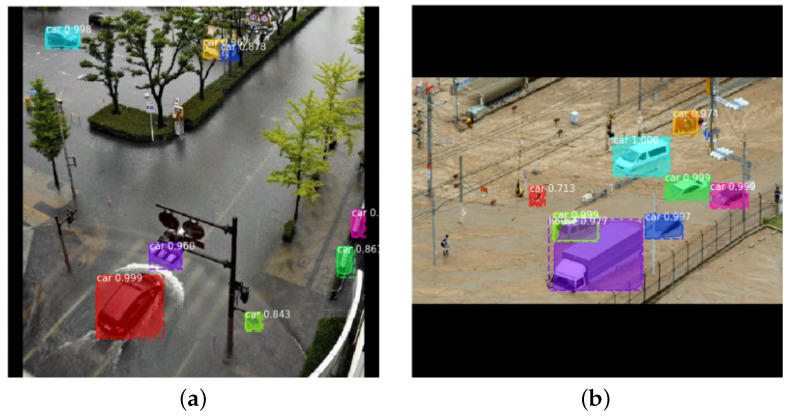
The output of the object detection module when APs: (**a**) 84.37% AP; (**b**) 76.00% AP.

**Figure 10:**
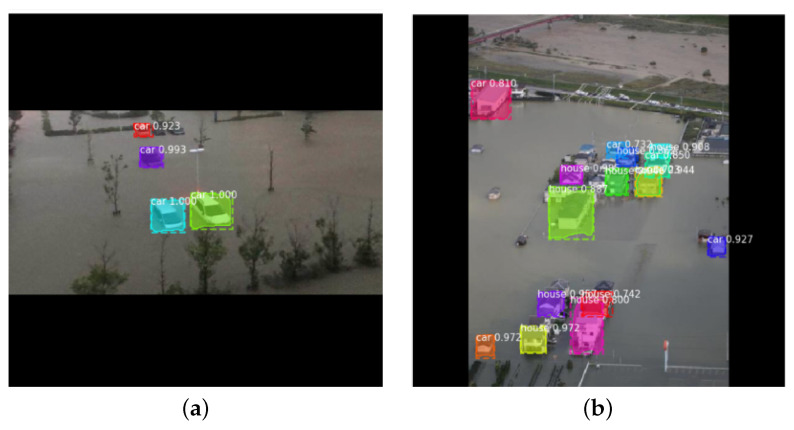
The output of the object detection module when APs: (**a**) 66.66% AP; (**b**) 22.50% AP.

**Figure 11:**
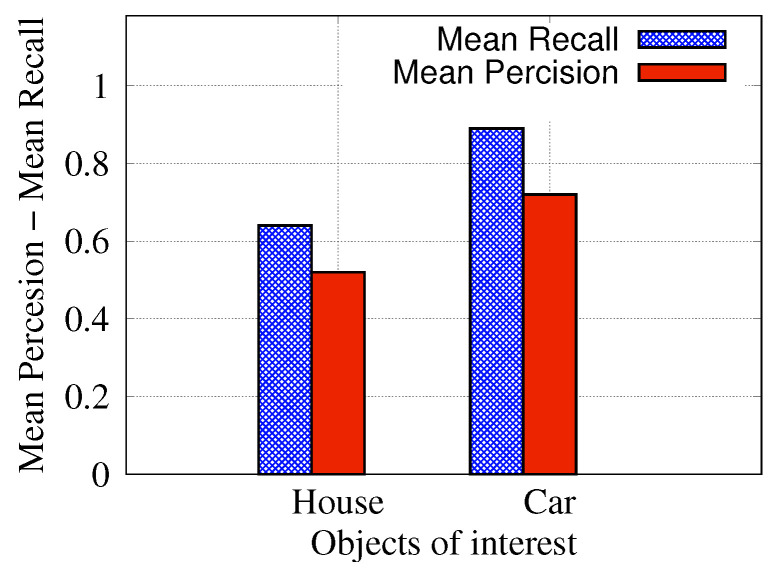
Precision and recall of each individual class.

Table 2 shows mAP, average precision and an average recall of the test images. It is worth noting that the AP results have high variations among individual images, and some examples are shown in Figure 9 and Figure 10. The recall value is higher than the precision value on average, which means that the model can detect most of the objects of interest even though a few other objects are also mistakenly detected without affecting the overall performance of the system. The recognition result for each class is shown in Figure 11. As can be observed from the figure, the detection results of the car class are better than those of the house class. This can be justified as the detection model has been pre-trained on the CoCo [25] dataset which does not include houses. Therefore, the obtained house detection results are only due to training the model from scratch on our small size dataset. It is worth mentioning that detection of every object (house or car) in the scene is not our end goal as water level (the end goal) can be estimated from a few objects in the scene. However, we expect higher house detection accuracy if more training images are provided, which is our ongoing work.

**Figure 12:**
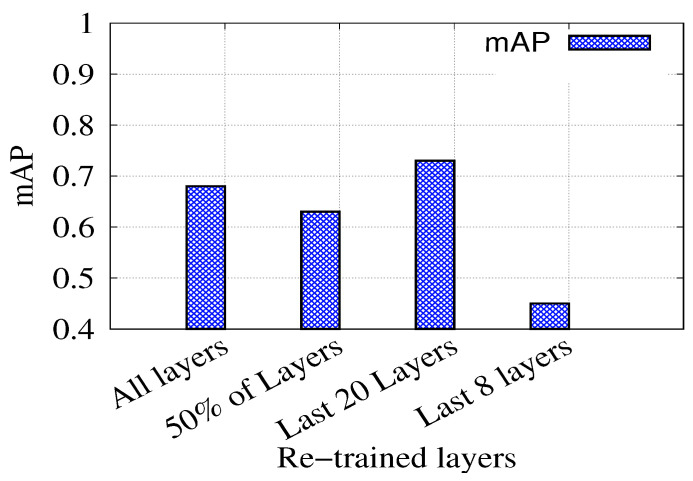
Effect of changing re-trained layers on the result.

Figure 12 shows the effect of changing the number of retrained layers on the Mask R-CNN model performance. The figure shows that the best accuracy was obtained by only re-training the last 20 layers. This can be attributed to the fact that the available data are not sufficient to re-train the whole model (i.e., all layers). On the other hand, re-training only the last few layers does not benefit the model because the representation at prior/earlier layers remains tightly coupled to the original dataset.

### 4.2. Water Level Estimation

For the water level estimation, 627 objects are considered. The distribution of the different classes and different water levels are shown in Table 3.

**Figure 13:**
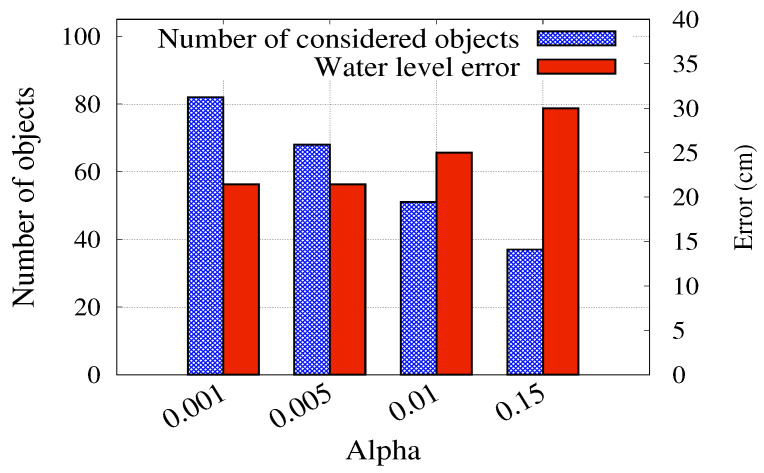
Effect of changing α on the system performance.

Data augmentation, described in Section 3.3, increases the amount of data to 1881 images of cropped objects. Then, 80% of them were used for training, and the remaining 20% were used for testing. The test data were further filtered by the policy described in Section 3.5. Figure 13 shows how the system performance is affected by the filtering threshold α that controls the size of the filtered-out images. The figure shows that a large α value increases the number of objects filtered out which leads to a loss of information (objects). Alternatively, a small α value allows very small objects leading to an increase in the computational cost with no advantage in the water level estimation. Therefore, an α threshold of 0.005 is selected to balance the trade-off between the estimation accuracy and the number of considered objects. As a result, our water level estimator achieves 57.14% accuracy with a mean water level error of 21.43 cm.

## 5. Conclusions

This research has highlighted the significance and challenges of automatic disaster management systems, as applied specifically to floods. Among various kinds of information, the water level has a significant contribution to the situation analysis and efficient and effective emergency response plans. The proposed system introduces a learning technique to determine a water level from the aerial top-view images, which is promptly available from the current drone technology. In particular, the system leverages a standard specification of submerged objects to identify the water level. It operates using a 2-step mechanism: top-view object detection and water level classification. For the first step, it applies a transfer learning technique on top of the *Mask R-CNN* network, and 0.73 mean average precision is achieved. For the water-level classification, the system obtains a 21.43 cm error, which is acceptable for flood situation analysis. We are now working with a company that has its top-view images repository for various types of disasters. With more data, we plan to improve accuracy and develop a real-time detection system.

## Figures and Tables

**Table 1:** Selected values of the mask R-CNN hyperparameters.

Learning Rate	#ROIs	Training Layer	Input Image Size	Epochs
0.001	128	Heads Layer	512 × 512 × 3	100

**Table 2:** Object detection result.

mAP	Average Precision	Average Recall
0.73	0.71	0.79

**Table 3:** The distribution of the considered objects and water levels.

Total	House	Car	0 m	0.5 m	1 m	1.5 m	2 m	2.5 m	3 m
627	366	261	120	207	134	77	7	81	1

## Data Availability

Data sharing not applicable.
